# The Pivotal Role of Major Chromosomes of Sub-Genomes A and D in Fiber Quality Traits of Cotton

**DOI:** 10.3389/fgene.2021.642595

**Published:** 2022-03-24

**Authors:** Abdul Razzaq, Muhammad Mubashar Zafar, Arfan Ali, Abdul Hafeez, Faiza Sharif, Xueing Guan, Xiaoying Deng, Li Pengtao, Yuzhen Shi, Muhammad Haroon, Wankui Gong, Maozhi Ren, Youlu Yuan

**Affiliations:** ^1^ State Key Laboratory of Cotton Biology, The Ministry of Agriculture, Institute of Cotton Research, Chinese Academy of Agricultural Science, Anyang, China; ^2^ Key Laboratory of Biological and Genetic Breeding of Cotton, The Ministry of Agriculture, Institute of Cotton Research, Chinese Academy of Agricultural Science, Anyang, China; ^3^ Institute of Molecular Biology and Biotechnology, The University of Lahore, Lahore, Pakistan; ^4^ FB Genetics Four Brothers Group, Lahore, Pakistan; ^5^ University Institute of Physical Therapy, The University of Lahore, Lahore, Pakistan; ^6^ Zhejiang University, Hangzhou, China; ^7^ School of Biotechnology and Food Engineering, Anyang Institute of Technology, Anyang, China; ^8^ National Key Laboratory of Crop Genetic Improvement, Huazhong Agricultural University, Wuhan, China; ^9^ Zhengzhou Research Base, State Key Laboratory of Cotton Biology, Zhengzhou University, Zhengzhou, China

**Keywords:** fiber quality, cotton fiber development, chromosome 7 significance, *Gossypium* species, stable QTLs, SNPs

## Abstract

Lack of precise information about the candidate genes involved in a complex quantitative trait is a major obstacle in the cotton fiber quality improvement, and thus, overall genetic gain in conventional phenotypic selection is low. Recent molecular interventions and advancements in genome sequencing have led to the development of high-throughput molecular markers, quantitative trait locus (QTL) fine mapping, and single nucleotide polymorphisms (SNPs). These advanced tools have resolved the existing bottlenecks in trait-specific breeding. This review demonstrates the significance of chromosomes 3, 7, 9, 11, and 12 of sub-genomes A and D carrying candidate genes for fiber quality. However, chromosome 7 carrying SNPs for stable and potent QTLs related to fiber quality provides great insights for fiber quality-targeted research. This information can be validated by marker-assisted selection (MAS) and transgene in *Arabidopsis* and subsequently in cotton.

## Introduction

Cotton is a major cash crop cultivated worldwide (Nadeem et al., 2021). It belongs to the *Hibiscus* or mallow family (Malvaceae) (Wendel et al., 2009). Cotton as a natural fiber is considered a potential cash crop mainly for the textile industry. The demand of the textile industry is increasing due to the high consumption rate and considerable marketability. It is planted over 80 countries in the world. Of the 52 species of *Gossypium* known to date, only four species, i.e., *G*. *hirsutum, G. barbadense, G. arboreum,* and *G. herbaceum* are mostly cultivated around the globe (Fryxell, 1992; Kunbo et al., 2018). *Gossypium hirsutum* has considerable adaptability to different environments and better yield compared with the other cultivated species, covering almost 95% needs of the global population (Chen et al., 2007). Allotetraploid cotton was originated by the hybridization of extinct progenitors of *G. herbaceum* (A1) or *G. arboreum* (A2) and *G. raimondii* (D5) (Wendel, 1989). Upland cotton (*G. hirsutum*) was first domesticated about 4,000–5,000 years ago. Later on, evolutionary studies and domestications divided the upland cotton into three groups, namely, improved modern cultivars, land races, and semi-wild (Fang et al., 2017). *Gossypium hirsutum* belonging to semi-wild is recognized with seven races (Hutchinson, 1951). The genus *Gossypium* consists of 45 diploid (2*n* = 2× = 26) and seven tetraploid (2*n* = 4× = 52) species. The diploid species are clustered into eight genomic groups (A–G and K) (Wang M. et al., 2019). Interspecific hybridization generated allotetraploids in America, and subsequently, polyploidization of A and D sub-genomes took place. Approximately, 1–2 million years ago, these two sub-genomes reassembled during the geographical change. Probably, *G. herbaceum* (A1) and *G. arboreum* (A2) are the ancestral donors of genome A, while *G. raimondii* (D5) is the donor of genome D (Hu et al., 2019). Normally, species of A genome bring out spinnable fibers, while D genome species are not cultivated. Approximately, worldwide, more than 90% annual cotton is met by *G. hirsutum* (AD)_1_-Upland cotton (USDA, 2019b).

Generally, the fiber length and strength are principal among all other fiber quality traits including fiber uniformity and micronaire (Yang et al., 2016). Fiber quality traits are paramount for the spinning technology in the textile industry, and they influence the fiber processing and dyeing consistency (Rodgers et al., 2017). Both fiber quality and yield are complex quantitative traits. The fiber yield trait is controlled by multiple genes and is much influenced by epigenetics, whereas the fiber quality is mainly controlled by the gene additive effect and is less affected by the environment (Ming-bao et al., 2008; Zhang et al., 2017).

Although fiber development studies are being carried out for many years, the molecular bases are still hardly known (Zhang B. et al., 2013). Molecular markers are potential tools for QTL mapping and dissecting the molecular processes of fiber quality characteristics. At least, 1,075 and 1,059 QTLs from 58 to 30 research studies, respectively, have been suggested with intraspecific and interspecific crosses (Said et al., 2015). These can further be validated by gene cloning to dissect the processes of complex traits. Moreover, functional studies explain the underlying molecular processes of fiber development in upland cotton.

Genome sequencing provides recent insights into the genome of cotton and has explored significant variations among the species or cultivars. The availability of the whole-genome sequence provides comprehensive information of fiber and fiber-related characteristics. The initial research study was started with the closest diploid progenitor *G. raimondii* (D_5_) and *G. arboreum* (A_2_) (Zhang et al., 2015a) which expanded to other tetraploid species *G. barbadense* (AD)_2_ and *G. hirsutum* (AD)_1_ (Hu et al., 2019). Allotetraploid species are economically significant in natural fiber production worldwide. *Gossypium hirsutum* produces better fiber yield, whereas *G. barbadense* even under harsh environmental conditions, can produce superior quality fiber over a process of evolution. The genetic foundations of these interspecies divergences are unrevealed. The information generated on the genome assembly and sequencing enables the breeders to revamp the fiber quality and resilience to variable environments (Yang et al., 2019). Reverse genetic techniques such as gene silencing (Kuttenkeuler and Boutros, 2004), targeted gene disruption by homologous recombination (Iida and Terada, 2004), insertional mutagenesis (Stemple, 2004), and chemical mutagenesis (Gilchrist and Haughn, 2005) play a vital role in identifying the function of the gene phenotypically. The genome sequencing information can help breeders to better understand the variations in the genome structure of chromosomes over-time, producing better fiber yield and quality. Based on this genetic information, the studies can be expanded for transgenes using recent molecular approaches. This comprehensive review provides the significance of different stable QTLs and candidate genes located on different regions of the chromosomes, with a special insight of chromosome 7 in upland cotton exploring the potential for improved fiber quality and quantity.

## Cotton Fiber Structure and Composition

Primarily, the cotton fiber is composed of 88.0–96.5% of cellulose (Goldwaith and Guthrie, 1954); however, a secondary cell wall is solely made of cellulose. The non-cellulosic components are located either on the cuticle or inside the lumen. They include proteins (1.0–1.9%), waxes (0.4–1.2%), pectins (0.4–1.2%), inorganics (0.7–1.6%), and other (0.5–8.0%) substances (Fan et al., 2005). The composition of cotton fiber undergoes dynamic changes throughout its development. During the early stage of 0–2 DPA, 1) xyloglucan is depleted, and the outer sheath is developed 2) an epitope (1–6)- β-D-galactan is lost carrying arabinose (Vaughn and Turley, 1999; Bowling et al., 2011), and 3) synthesis of the cotton fiber middle lamella (CFML) begins. This outer thin primary cell wall augmented with fucosylated and non-fucosylated xyloglucan and homogalacturonan helps connect adjacent fibers into tissue-like bundles, assembling into firmly packed fibers within the confined boll space (Singh et al., 2009). The inner primary wall of the cotton fiber contains ∼22% crystalline cellulose fibrils surrounded by pectin and xyloglucan (Singh et al., 2009). In the cotton fiber, the glycome profiling of the fiber shows the presence of xylan and acetylated mannan (Pattathil et al., 2010). During the transition phase of primary and secondary cell wall deposition, degradative enzymes break down the CFML to release the fibers individually—a phenomenon in which primary wall-related sugars are lessened, cell wall hydrolases are upregulated, pectin is reduced, and xyloglucan molecular mass, as secondary wall synthesis, begins (Gou et al., 2007; Singh et al., 2009).

**FIGURE 1:**
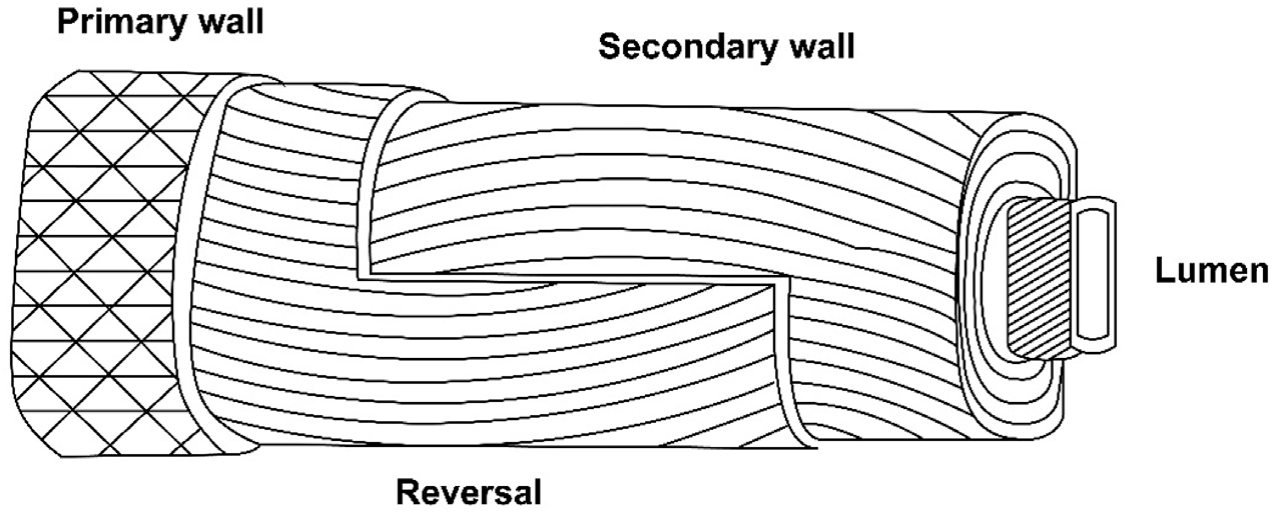
Generalized structure of the cotton fiber (M. Dochia and Z. Roskwitalski, 2012).

A unique winding of cell wall layers occurs during the transition of primary and secondary walls. This unique winding is similar to the S1 layer in the wood fiber (Seagull, 1993) (Figure 1). During the same transition phase rearrangement of microtubules, a decline in the respiration rate and change in the concentration of metabolic sugars occur, whereas the cellulose synthesis rate increases to ∼35% (w/w), and the CFML undergoes degradation (Singh et al., 2009). Although, wall thickening is minimal at this stage, fiber strength increases significantly, seemingly because of differentially placed cellulose microfibrils in the layer. So the secondary wall of cotton fiber becomes 3–6 μm thick (Hinchliffe et al., 2011).

## Cotton Fiber Developmental Biology

The cotton fiber is a seed-borne epidermal trichome. It is made of thickened and elongated single cells. The turgor pressure in the cell causes an expansion in the cotton fiber. (Cosgrove, 1997). Fiber development occurs 3 days before and after anthesis coupled with enlargement of epidermal cells and protrusion of trichomes. Both the lint percentage and lint index are positively associated with the density of trichome protrusion (0 DPA) and fiber initials (1 DPA) in some cultivars of *G. hirsutum* (Li et al., 2009). Elongation of the fiber starts within a period of 2DPA to 20DPA, followed by initiation. The twisting of elongating fibers generates several fibers (Haigler et al., 2012). Cellulose synthesis takes place at 10–16 DPA, stimulating the closing of plasmodesmata that is paramount for fiber elongation. At the cellular level, the mechanism of the *GhVINI* gene expression (Wang et al., 2010), expression of pectin biosynthesis genes (Pang et al., 2010), expression of expansins (Li et al., 2016a), and aquaporin proteins and their corresponding genes is stimulated, which plays a key role in development of cotton fibers (Liu et al., 2008). Additionally, during cotton fiber differentiation, DNA methylation has been reported, which is related with the modification of chromatin (Wang et al., 2016).

**FIGURE 2:**
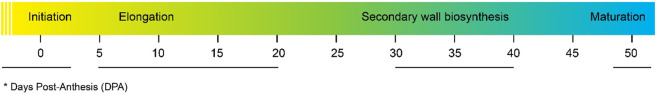
Cotton fiber development stages at various DPA.

Fiber elongation stops during the transition of cell elongation to cell wall biosynthesis making the secondary cell wall (SCW) thickened with the upregulation of SCW genes. The rate of synthesis of cellulose, the orientation of its fibrils, and the individual length of the cellulose chain synthesized by cellulose synthase directly deals with the thickness of the SCW (Betancur et al., 2010). In sclerenchymatous cells, several flavonoids and lignin biosynthesis pathways are involved in the regulation of SCW synthesis (Zhao and Dixon, 2011) (Figure 2).

The single cell expression system provides greater insight on understanding the mechanism of fiber development (Haigler et al., 2009). During cell wall thickening, the primary cell wall (PCW) feature of *Arabidopsis* leaf trichomes relates to the SCW of the cotton fiber (Betancur et al., 2010).

Four stress-related genes were identified, and the expression pattern was analyzed. The phenotypic data represented that abiotic stress affects the overexpression of the cotton genes in *Arabidopsis* (Li et al., 2020). In another study, it was reported that the treatment of sodium chloride enhances the gene expression for ∆-pyrroline-5-carboxylase synthetase, sucrose synthase, cellulose synthase A, and sucrose phosphate synthase in leaves and fibers of the transgenic plants (Kesanakurti et al., 2010). Furthermore, the study reported that salt stress shows diverse responses over *O*-methyltransferase genes during fiber development and may be involved in the salt tolerance of *Gossypium* species (Hafeez et al., 2021). This characteristic provides a roadmap to further validate the functions of related genes.

## Role of Major Genes in Fiber Quality Development Across the Whole Genome

**FIGURE 3:**
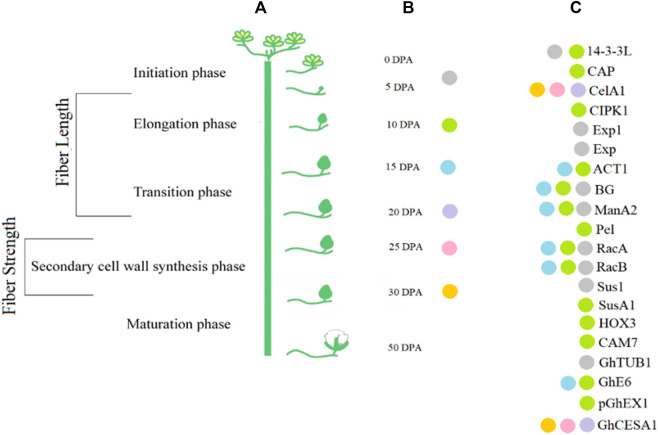
Interaction of candidate genes involved in regulation of fiber quality traits.

Transcript profiling is important in providing information about various phases of fiber development. Transcription factor (TF) families such as MYB, C_2_H_2_, and WRKY are involved in fiber initiation (Zhang et al., 2010). Different genes are differentially expressed at earlier stages of fiber initiation and development in different species of *Gossypium* (Samuel Yang et al., 2006; Zhang et al., 2010). Plant hormones also play a key role in fiber development. For example, *GhPIN*-mediated auxin transport is involved in the ovule-specific suppression of the *GhPIN* gene, which reveals its involvement in fiber initiation (Zhang J. et al., 2016). A candidate gene *Pel* (Accession ID: DQ073046) on chromosome 7 has been reported, which is involved in the breakdown of de-esterified pectin and in the fiber elongation process (Bai et al., 2014). Some of the potential genes with their accession numbers are listed in Table 1. A schematic diagram represents the involvement of genes associated with the fiber strength and length (Figure 3).

**TABLE 1:** Major genes for fiber development across the whole genome of *G. hirsutum*.

Gene	Accession ID	Protein/Enzyme	Primary function of gene	Reference
*CEL*	AY574906	Endo 1,4-β-glucanase	Plays a major role in secondary wall development	Pear et al. (1996)
*CIPK1*	EF363689	CBL-interacting protein kinase	Regulates fiber elongation	Gao et al. (2007)
*BG*	DQ103699	β-1,4-Glucanase	Regulates biosynthesis of the secondary cell wall	Ma et al. (2006)
*RacA*	DQ667981	Small GTPase	Regulates fiber development	Li et al. (2005b)
*LTP3*	AF228333	Lipid transfer protein gene	Regulates primary cell wall synthesis	Liu et al. (2000)
*CAM7*	TC232366	Calmodulin protein	Regulates the cotton fiber length	Cheng et al. (2016)
*GhE6*	BM356398	Fiber protein E6	Involved in biosynthesis of fiber	John and Keller, (1996)
*GhCESA1*	U58283	Cellulose synthase A catalytic subunit 8	Regulates secondary wall synthesis	Pear et al. (1996)
*14-3-3L*	DQ402076	14-3-3l	Expresses during fiber development	Shi et al. (2007)
*CelA3*	AF150630	Cellulose synthase catalytic subunit	Regulates cellulose synthesis	Zhang et al. (2012)
*Exp*	DQ060250	Expansin	Involved in the fiber length	Zhu et al. (2012)
*Pel*	DQ073046	Pectate lyase	Responsible for fiber elongation	Bai et al. (2014)
*Sus1*	U73588	Sucrose synthase	Involved in fiber initiation and elongation	Ruan et al. (2003)
*HOX3*	107904747	Homeodomain protein	Differentially expressed for fiber elongation	Wang et al. (2004)
*GhTUB1*	AF487511	Beta tubulin	Associated with fiber elongation	Zhang et al. (2003)
*GhGIcAT1*	AY346330	Glycuronosyl transferase-like protein	Associated with non-cellulosic cell wall synthesis	Wu et al. (2006)
*cesA4*	817092	Cellulose synthase A catalytic subunit 3 UDP-forming- like protein	Regulates post-translational modifications	Polko and Kieber, (2019)
*GhGIcAT1*	AY346330	Glycuronosyl transferase-like protein	Associated with non-cellulosic cell wall synthesis	Wu et al. (2006)
*CAP*	AB014884	Adenylyl cyclase-associated protein	Expresses during early development of fiber	Kawai et al. (1998)
*CelA1*	GHU58283	Cellulose synthase	Regulates cellulose synthesis	Chen and Burke, (2015)
*Exp1*	DQ204495	α-Expansin 1	Involved in fiber length	Shi et al. (2006)
*ACT1*	AY305723	Actin 1	Differentially expressed during fiber elongation	Li et al. (2005a)
*ManA2*	AY187062	β-Mannosidase	Involved in fiber development	Zhu et al., 2011
*RacB*	DQ315791	Small GTPase	Involved in fiber quality parameters	Li et al. (2005b)
*pGhEX1*	AF043284	Expansin-A4-like	Vital in fiber elongation	Orford and Timmis, (1998)
*CelA1*	AF150630	Cellulose synthase catalytic subunit	Development of cotton fiber	Zhu et al. (2012)
*SusA1*	HQ702185	Sucrose synthase	Expresses during fiber elongation	Jiang et al. (2012)

Note: Some of the key genes listed in Table 1 are taken from the study by Ijaz et al. (2019).

Molecular breeding has indicated some potential genes with a notable role in the fiber quality, yet they need to be verified through functional genomics or other advanced molecular techniques including the transgenic technology (Table 2). Two major genomic regions (MGR1 and MGR2) of upland cotton on chromosome Dt7 were reported with four potential candidate genes corresponding to the fiber length and fiber strength. It was found that MGRI regions contain three putative candidate genes qualified for the fiber length (FL), while the MGR2 region corresponding to *CotAD_35088* qualified for both fiber length and fiber strength (Su et al., 2016). Similarly, a CSSL line (MBI7747 and MBI7561) was established with a cross of high-yielding CCRI45 (*G. hirsutum*) and high-quality fiber Hai1 (*G. barbadense*) species, and potential candidate genes were found (Lu et al., 2017).

**TABLE 2:** Fiber quality development genes across the whole genome of *G. hirsutum*.

Gene name	Chromosome No.	Function	Reference
*GhRBB1*	A07	Regulates fiber development	Majeed et al. (2019)
*ADF1*	D03	Regulates development of fiber quality	Zhang et al. (2007)
*CotAD_35088*	D07	Associated with fiber quality parameters	Su et al. (2016)
*GhMYB1*	A09	Associated with fiber development	Loguercio et al. (1999)
*GhTCP5*	D12	Involved in fiber development	Loguercio et al. (1999)
*CotAD_22825*	D07	Associated with the development of cotton fibers	Su et al. (2016)
*GhMYB4*	A11	Downregulation of fiber elongation	Loguercio et al. (1999)
*GhMYB4*	A03	Regulates development of cotton fiber	Loguercio et al. (1999)
*GhCPC*	A11	Associated with fiber quality development	Liu et al. (2015b)
*GhTCP20*	D12	Plays a vital role during fiber initiation	Li et al. (2017)
		Upregulation of fiber elongation	
*GhMYB5*	A05	Plays a vital role in fiber development	Loguercio et al. (1999)
*Gh_D03G0889*	D03	Plays a vital role in protein Kinase	Diouf et al. (2018)
*GhTCP15*	A13	Regulates fiber development	Li et al. (2017)
*CotAD_22823*	D07	Involved in the development of cotton fiber	Su et al. (2016)
*MYB60*	D03	Regulates development of the fiber quality	Kasirajan et al. (2018)
*GhTCP12*	D12	Expresses during secondary cell wall deposition	Li et al. (2017)
*GhPAG1*	A09	Differentially expresses during development of cotton fiber	Yang et al. (2014)
*GhPIP2–4*	D12	Involved in the fiber length	Li et al. (2013)
*GhTCP11*	A09	Regulates fiber development	Li et al. (2017)
*Gh_D12G0093*	D03	Vital in protein Kinase	Diouf et al. (2018)
*GhPAG1*	A09	Associated with fiber development	Yang et al. (2014)
*GhTCP14*	D12	Regulates cotton fiber development	Li et al. (2017)
*RABB1C*	A07	RAB GTPase homolog B1C	Sun et al. (2017)
*RAB8*	D03	Associated with fiber quality development	Li and Guo, (2017)
*GhMYB4*	D01	Regulates fiber length	Loguercio et al. (1999)
*Lc1*	A07	Regulates development of dark fiber	Li et al. (2012)
*GhPRP3*	A11	Regulates biosynthesis of the cell wall	Wenliang et al. (2006)
*GAUT9*	D07	Galacturonosyl transferase 9	Sun et al. (2017)
*Gh14–3-3*	D01	Involved in fiber development	Zhou et al. (2015a)
*CotAD_22824*	D07	—	Su et al. (2016)
*GhMYB5*	A13	Downregulation of fiber development	Loguercio et al. (1999)
*GhTCP15*	D12	Plays a vital role in fiber elongation	Li et al. (2017)

During fiber development, differential expression studies help to find out fiber quality-related genes. For example, two recombinant inbred lines (RILs), differing in fiber quality, were taken from the intra-*hirsutum* population. The expression profiling differences and fiber quality-related genes were explored by RNA sequencing. It was noted that differentially expressed genes (DEG) *72/27*, *1,137/1,584*, *437/393*, *1,019/184*, and *2,555/1,479* were up/downregulated in lines (L1 high-fiber quality and L2 low-fiber quality) at 10, 15, 20, 25, and 30-day post-anthesis, respectively. Hence, 363 DEGs between L1 and L2 were co-localized in fiber strength QTL. Seven expression profiles were identified through short time-series expression minor (STEM) analysis. Kyoto Encyclopedia of Gene and Genomes (KEGG) and gene ontology (GO) annotations were performed to identify differences in the gene function associated with two lines L1 and L2 (Zou et al., 2019). The five modules of specific fiber development stages mainly for fiber quality were assessed by co-expression network analysis. It was noted that the relationship between hub and other genes can be revealed by the correlation network (Zou et al., 2019). Furthermore, transcriptome and QTL analysis of 780 differently expressed genes were studied (Wang et al., 2020).

## Significance of Current Technologies and Cotton Fiber Development

### Molecular Markers and the Linkage Map for Quantitative Trait Locus Identification

The high-density linkage map, germplasm evolution, and phylogenetic analysis are concomitant with the implication of molecular markers. These applications are highly helpful and efficient for the mapping of stable QTLs and recognition of genes related with development of fibers. Reinisch et al. (1994) developed the first genetic map using 705 RFLP markers that covered a length of 4,675 cm. Subsequently, different genetic maps were developed for fiber quality traits (Kohel et al., 2001). Molecular markers have become an integral component for the determination of fiber quality-related traits. The information can be applied across chromosomes of the *Gossypium* species to identify fiber quality-related QTLs and candidate genes.

Recombination inbred lines (RILs) and doubled haploids, F_2_, F_2:3_, and BC_1_ are primary populations (Cao et al., 2015; Islam et al., 2016a), while secondary populations are chromosome segment substitution lines (CSSLs) used in cotton (Islam et al., 2016b). The crossing of a donor and a recipient with several backcrossing with the recurrent parents develop CSSLs. The offspring and the recurrent parents have the same genetic background with a difference of single nucleotide or an Indel. CSSLs are considered ideal and efficient material for QTL analysis, fine mapping, and positioning cloning (Guo et al., 2013). The first CSSLs were developed by Esha and Zamir in 1994 and detected six QTLs in tomato related to its quality. Subsequently, these lines were successfully developed in maize, wheat, peanut, rice, soyabean, and other plants (Thomson et al., 2003; Zhou G. et al., 2015; He et al., 2015). The genetic map resulted from *G. hirsutum* and *G. barbadense* helps identify the QTLs related to fiber yield and quality. (Shi et al., 2015). It is reported that the development of CSSLs from TM-1 and Hai7124 increased the fiber quality (Wang et al., 2012). In another research, 51 QTLs were reported in 116 lineages of CSSLs using CCRI45 and Hai1 (Yang et al., 2009). The construction of chromosome segment substitution lines in *Gossypium* species is getting more attention to attain good fiber quality traits such as the fiber length and strength. This approach can be exploited across the economically important chromosome 7 to identify the most suitable stable QTLs and candidate genes.

### Sequencing in Cotton Fiber Development Using Advanced Next Generation Tools

The development of high density and precise QTL genetic linkage maps is due to the use of molecular markers associated with the availability of genome sequences of *G. raimondii*, *G. arboreum*, *G. hirsutum*, and *G. barbadense* (Yuan et al., 2016). Subsequently, high-throughput genotyping provided a deep insight for the better understanding of the genome-wide study. For example, cotton SNP63K and cotton SNP80K enabled the researchers to better understand the genetic mapping, genome, genomic selection, and genomic diversity within *Gossypium* (Cai et al., 2017). Different SNP markers can be deployed on common QTLs found under diverse environments for fine mapping and candidate gene identification (Su et al., 2016; Tan et al., 2018). This could be further boosted by the advancement of next-generation sequencing which includes restriction site-associated DNA (Jia et al., 2016). For example, a candidate gene *Pel* (Accession ID: DQ073046) on chromosome 7 has been reported which is involved in the breakdown of de-esterified pectin and during fiber elongation (Bai et al., 2014). Hence, by the use and exploitation of high-throughput sequencing techniques, as mentioned earlier, many differentially expressed genes, playing important roles in fiber quality traits, can be discovered.

### The Recent Developments in Genotyping and Quantitative Trait Locus Mapping

The rapid developments in bioinformatics and availability of the cotton genome have allowed to efficiently use SSRs and SNPs for effective genotyping and QTL mapping in cotton for both wild and domesticated species using intraspecific populations under multiple generations and diverse environments (Lacape et al., 2005; Wang et al., 2007; Zhang et al., 2009). For example, a recombinant inbred line (RIL) was developed by crossing cultivar Yumian1 and CA3084 (Ali et al., 2018). By using the SLAF-seq technology a genetic map consisting of 6,254 SNPs was developed with an average distance of 0.5 cm among the markers. A total of 95 QTLs for fiber quality were discovered, and among these, 55 were stable across multiple environments, whereas nine of the stable QTLs were found in three different environments (Ali et al., 2018). In another study, an interspecific cross between HS46 and MARCABUCAG8US-1-88 resulted in 188 F_8_ RILs (Li et al., 2016b). These were genotyped by the cotton SNP63K assays. During the cropping season under standard conditions, field trials were conducted at Sanya, Hainan province. Significant differences (*p* ≤ 0.05) were observed among the populations and environments. A linkage map consisting of 2,618 polymorphic SNP markers was developed with a density of 0.68 cM per marker. A total of 71 QTLs were found across 21 chromosomes with 16 as stable QTLs in two environments. On chromosomes 5, 9, 10, 14, 19, and 20, twelve various regions were identified. which are believed to be involved in the control of one hotspot or a group of characters (Li et al., 2016b).

Single nucleotide polymorphisms (SNPs) are significant in determining the population structure (Jain et al., 2014). The presence of SNPs in a coding region and regulatory sequence is of great importance because it may change the gene function (Hulse-Kemp et al., 2015b). Various techniques have been used for the discovery of SNPs in the cotton genome such as the single-copy sequence, BAC-end sequences, transcriptome-sequencing, reduced representation libraries (RRL) techniques, and genotyping by sequencing (GBS) (Hulse-Kemp et al., 2015a). The GBS is the most widely adopted method for the detection of SNPs in the crop due to being cost-effective and having rare chances of error (Majeed et al., 2019). However, re-sequencing is equally important for SNP studies (Bentley, 2006). SLAF-seq is also being used in many studies for uncovering the SNPs and Indel variations in the tetraploid cotton (Shen et al., 2017). Moreover, Pacific Biosciences Nanopore (Ashrafi et al., 2015) and Rosch Illumina 454 (Kumar et al., 2012) sequencing studies are meant for the determination of SNPs as long reads and as short reads, respectively, which can be performed with these techniques.

GWAS was performed on a panel of 355 accessions of upland cotton (Su et al., 2016). In this experiment, two major genomic regions such as MGR1 and MGR2 were deployed. A significant number of SNPs such as 16, 10, and 7 were determined related to fiber length, fiber strength, and uniformity on chromosome Dt7, respectively. This discovery of SNPs detected four candidate genes for the fiber length with different SNP loci (Su et al., 2016) meant for membrane protein, B3 domain-containing protein, membrane protein, and pentatricopeptide repeat-containing protein, respectively (www.cottonfgd.org). In another genome-wide association study, a total of 10,511 SNPs were distributed across 26 chromosomes of cotton, and only 46 significant SNPs relating to fiber quality were detected (Sun et al., 2017). Furthermore, these SNPs were scattered across 15 chromosomes, and 612 unique candidate genes were detected. Most of the candidate genes were found to be related to polysaccharide synthesis, signal transduction, and protein localization. In this study, on chromosomes Dt11 and At07, two haplotypes of fiber strength and length were identified. By combining transcriptome analysis and GWAS, 120 and 163 fiber quality-related genes were discovered, and out of these, 19 genes were screened as promising genes (Sun et al., 2017).

In a subsequent study, the genome-wide association revealed 209 and 139 QTNs for yield components and fiber quality traits, respectively (Liu et al., 2018). Among the total QTNs, 57 were found as stable in two environments. The candidate genes observed through 57 stable QTLs were analyzed with those related with QTN, and it was found that 35 candidate genes were common in both. However, it was also reported that four genes were pleiotropic among these common candidate genes (Liu et al., 2018). In another investigation, a GWAS-based high-density cotton SNP80K was used for the identification of genes related to fiber quality (Dong et al., 2019). Only 30 SNPs were found from 408 cotton accessions related with fiber quality traits across six environments. Seven loci were found to be the same among these SNPs with 128 candidate genes predicted in the vicinity of 1 Mb. Two major genome regions (GR1 and GR2) on chromosomes A07 and A13 associated with varied fiber qualities were found in multiple environments. Among these, 22 candidate genes were annotated using RNA-seq, and of these, 11 were expressed at different developmental stages (Dong et al., 2019).

As discussed earlier, the SNP discovery associated with fiber quality traits has opened new horizons in biotechnology. A single SNP in a coding region can alter the gene function. With the growing demand for cotton and challenges this crop cultivation, processing and marketing are facing, different protocols and assays are being extensively employed for the determination of stable QTLs, QTNs, and SNPs linked with the fiber quality over the genome of tetraploid cotton. It was experienced that like others, chromosome 7 has a fundamental role for fiber quality development. The role of SNPs for the detection of QTLs associated with the fiber quality can be seen in Table 3.

**TABLE 3:** Role of SNPs for the determination of QTLs of fiber quality traits across chromosome 7 in *G. hirsutum*

Cotton species	Source of the genotype	Genotyping method	No. of experimental years/ Location/ Environment	Trait	No. of QTLs/QTNs/SNPs	Chromosomal location	Reference
*G. hirsutum*	Magic population, fifth-generation	GBS	Four Environments	Superior fiber quality	6071 SNPs	A07	Islam et al. (2016c)
*G. hirsutum*	F_2_ population	dCAPS	1	Short fruiting branch gene	1 SNP locus	D07	Zhang et al. (2018)
*G. hirsutum*	555 RILs and 11 parents	GBS	4 Years	Fiber quality	6 071 SNPs	A07	Islam et al. (2016c)
			Two locations		86 QTLs		
*G. hirsutum*	160 early-maturing cotton accessions	SLAF-seq	Two environments	—	70 QTNs	D03, A05, D11	Su et al. (2018)
*G. hirsutum* × *G. barbadense*	168 F_2_ population	GBS	1	Nulliplex-branch gene *(gb_nb1)*	42 SNPs	16(D07)	Chen et al. (2015)
*G. hirsutum*	419 accessions	GBS	12 Environments	Fiber-related traits	3 665 030 total SNPs	A07, A10, D03, D11	Ma et al. (2018)
					7 383 unique SNPs		
*G. hirsutum*	F2 population	SLAF-seq	Multiple environments	sucrose synthesis	2 QTLS	D03	Zhang et al. (2019a)
*G. hirsutum*	169 accessions	Cotton SNP 80 K array	2 Years	Fiber quality traits	342 QTNs	A01, A06, A07, D01, D12, D13	Li et al. (2018)
			Two locations				
*G. hirsutum*	F2:3 lines	GBS	1	Plant height, the height of fruiting, branch node, and number of vegetative shoots	17 QTLs	03, 04, 05, 07, 09, 17, 19, 23, 25	Qi et al. (2017)
*G. hirsutum*	355 accessions	SLAF-seq	Four environments	Fiber quality traits	33 SNPs	D07	Su et al. (2016)
*G. hirsutum*	169 accessions	Cotton SNP 80 K array	2 Years,	Earliness	49 650 SNPs, 29 Significant SNPs	A06, A07, A08, D01, D02, D09	Li et al. (2018)
*G. hirsutum*	355 accessions	SLAF-seq	Four environments	Fiber quality traits	33 SNPs	D07	Su et al. (2016)

It is much evident from the aforementioned studies conducted across the whole genome of *G. hirsutum* L. that different sequencing techniques such as SSR, GBS, SLAF-seq, CottonSNP63K, and CottonSNP80K significantly help identify the stable QTLs and significant SNPs across different chromosomes and reveal their importance in fiber quality development. In Table 1, 2, different genes related with fiber quality development are reported across the whole genome of cotton. It is depicted from the information mentioned previously and in Tables 1,2 that chromosomes 3, 7, 9, 11, and 12 of sub-genomes A and D are harboring most of the genes associated with fiber quality development at different stages in cotton. In this study, we will continue with the importance of chromosome 7 as various studies conducted in our laboratory group have shown different stable QTLs and differentially expressed candidate genes associated with fiber quality traits. The information mentioned in Tables 3 and 4 reveals the importance of chromosome 7 containing hotspots related to cotton fiber quality.

**TABLE 4:** Prospective QTLs of fiber development located on chromosome 7 in *G. hirsutum* using SSR markers.

Trait	QTL	Flanking marker	Population	References
Fiber micronaire (FM)	qFM-C7-1	DC40182	F_2:3_	Chen et al. (2018)
—	—	DPL0757-BNL1604, DC40182, SWU8784, and SWU8789	F_2_ and F_2:3_	Fang et al. (2017)
—	qFM-C7.1, qFM-C7.2	DC40182 and DPL0852	F_2_ and F_2:3_	Chen et al. (2018)
—	—	DPL0757, BNL1604, and DC40182	F_2_, F_2:3_, and RILs	Sun et al. (2012)
—	—	DPL0757 and BNL1604	—	
—	—	DPL0757 and BNL1604	F_2_ and F_2:3_	Tan et al. (2015)
Fiber uniformity (FU)	qFU-C7.1	DPL0757 and BNL1604	F_2_ and F_2:3_	Tan et al. (2015)
—	—	DC40182 and DPL0852	F_2_ and F_2:3_	Chen et al. (2018)
—	—	NAU 2002, CGR6381, NAU1048, and CICR6381	BC_5_F_2_ and BC_5_F_2:3_	Li et al. (2019)
Fiber length (FL)	qFL-C7-1	DC40182	RIL and F_2:3_	Chen et al. (2018)
—	—	DPL0757-BNL1604, DC40182, SWU8784, and SWU8789	F_2_ and F_2:3_	Fang et al. (2017)
—	—	SHIN1447 and DPL0757	F_2_ and F_2:3_	Wang et al. (2015)
—	—	DC40182 and DPL0852	F_2_ and F_2:3_	Chen et al. (2018)
—	—	DPL0757, BNL1604, and DC40182	F_2_, F_2:3_, and RILs	Sun et al. (2012)
—	—	DPL0757 and BNL1604	F_2_ and F_2:3_	Tan et al. (2015)
Fiber elongation (FE)	qFE-C7-1	SHIN1447 and DPL0757	F_2_ and F_2:3_	Wang et al. (2015)
—	—	DC40182 and DPL0852	F_2_ and F_2:3_	Chen et al. (2018)
—	—	DPL0757 and BNL1604	F_2_ and F_2:3_	Tan et al. (2015)
Fiber strength (FS)	qFS-C7-1	DC40182	F_2:3_	Chen et al. (2018)
—	—	DPL0757-BNL1604, DC40182, SWU8784, and SWU8789	F_2_ and F_2:3_	Fang et al. (2017)
		SWU2662 and SWU2707	F_2_ and F_2:3_	Paterson et al. (2012)
—	—	SHIN1447 and DPL0757	F_2_ and F_2:3_	Wang et al. (2015)
—	—	DC40182 and DPL0852	F_2_ and F_2:3_	Chen et al. (2018)
—	—	DPL0757, BNL1604, and DC40182	F_2_, F_2:3_, and RILs	Sun et al. (2012)
—	—	DPL0757 and BNL1604	F_2_ and F_2:3_	Tan et al. (2015)
—	—	SWU0473, PGML01916, and NAU1085	BC_5_F_3:5_	Song et al. (2017)
—	—	NAU 2002, CGR6381, NAU1048, and CICR6381	BC_5_F_2_ and BC_5_F_2:3_	Li et al. (2019)
—	—	CGR6381	BC_7_F_2_	Zhang et al. (2019b)

## Quantitative Trait Loci of Fiber Quality Development Across Chromosome 7

As mentioned earlier, the fiber length and fiber strength are major economic traits in cotton production. The QTL studies under different environments and across multiple generations allow scientists to identify common QTLs for marker-assisted selection (MAS). For example, by deploying the SSR markers, several QTLs were identified for fiber length, fiber strength, fiber micronaire, and fiber elongation (Tan et al., 2015). In another study, stable QTLs for fiber length and micronaire on chromosome 7 were detected, which could be further validated for fine mapping and identification of candidate genes (Ali et al., 2018). The QTL clusters of fiber quality under different environments were also detected on chromosomes c4, c7, c14, and c25 (Jamshed et al., 2016). The superior alleles of fiber quality were introgressed from *G. barbadense* to *G. hirsutum* which were found to be linked in five QTL clusters associated with potential candidate genes for fiber development (Chen et al., 2018).

Fiber quality traits are affected by the environment in which the crop is subjected to grow. However, improved lines always perform well compared with corresponding recurrent parent lines in each environment (Tan et al., 2015). As discussed previously, the fiber length and strength are economic traits, and they correlate positively with each other. Different studies have been performed in different environments for the identification of stable QTLs of fiber quality, especially FS and FL across the whole cotton genome. It has been shown by various findings that chromosome 7 contributes potentially in fiber development. For example, a major stable QTL of fiber strength qFS07.1 was identified on chromosome 7 with favorable alleles contributed by the cultivar Yumian1 in five different environments (Tan et al., 2015). A fine mapped population was produced for qFS07.1 by crossing CCRI35 and RILs with Yumian1 allele using SSR primers according to *G. raimondii* and *G. arboreum* genomes (Paterson et al., 2012; Li et al., 2014). Some extensive studies across chromosome 7 have reported some stable QTLs for fiber quality traits using different mapping techniques; they have been described as follows in Table 4.

In upland cotton (*G. hirsutum*), a three-parent composite population was used to identify stable QTLs of fiber quality traits on chromosome 7 between HAU1367 and HAU2282 (Zhang et al., 2012). Another study using F_2_ and F_2:3_ family lines derived from Luyuan (LY343) and Lumianyan (LMY22) was conducted, and some fiber quality-related traits were identified on chromosome 7 flanked by SSR markers (Wang et al., 2015). The upland cotton was crossed between 0 and 153 and sGK9708 for F_2_, F_2:3_ and RIL population, which allowed in identifying fiber quality traits across chromosome 7 using markers DPL0757, DC40182, and BNL 1604 (Sun et al., 2012; Fang et al., 2017). A series of improved lines (BC_2_F_3_) of super fiber quality were developed using marker-assisted selection (MAS) (Cao et al., 2015). One of the improved lines, 3,326–7, was transferred to an introgression line (ILo88-A7-3) derived from a cross of *G. hirsutum* acc. TM-1 and *G. barbadense* cv. Hai7124. It was found that this line had a consistency of producing super quality fiber properties. It was further revealed by substitution mapping of 229 BC_3_F_2_ recombinants with 207 BC_3_F_3_ and BC_3_F_4_ lines that qFLS-chr.7 and qFS-chr.7 were anchored to the same target position with an interval of 0.36-cm using markers NAU3735 and NAU845, while qFM-chr.7 was mapped with an interval of 0.44 cm using SSR markers. This positive relationship of tightly linked QTLs demonstrates their contribution to fiber quality development, especially in fiber strength and fiber length (Cao et al., 2015).

CSSLs are used as an ideal material for the identification of genetic effects. In a study, a material of CCRI45 (*G. hirsutum*) was crossed with Hai1 (*G. barbadense*) and MBI9915, and the CSSL was selected at BC4F3:5 (Li et al., 2019). A total of 2,292 SSR markers were applied which covered the whole tetraploid cotton genome. A total of 129 QTLs were detected including 103 for fiber quality and 17 for fiber yield across 17 chromosomes with a phenotypic effect of 0.85–30.35%. Out of the total, 39 were stable, 53 were common, and 76 were new with a phenotypic variation of 30.2, 41.1, and 58.9%, respectively, with 86 of favorable effects on related traits. It was also noted that more QTLs were distributed across the Dt sub-genome than the At sub-genome. Out of 25 stable QTL clusters detected across 22 chromosomes, only six introgressed segments with a special focus on Seg-A07-2 were found as important candidate chromosome regions for fiber quality (Li et al., 2019).

In another investigation, a new CSSL material, MBI9915, was obtained by crossing CCRI36 (*G. hirsutum*) with Hai1 (*G. barbadense*) (Song et al., 2017). An F_2_ population of 1,537 individuals was constructed by crossing MBI9915 and CCRI36, and 347 individuals from the F_2_ population were randomly selected for the study of the genetic effects of the introgressed chromosome segments. A total of 18 and six QTLs were detected for fiber quality and fiber yield, respectively, in two segregating populations with the cumulative phenotypic variation of 0.81–9.51% on chromosome 7. Among these QTLs, six were detected consistently, as detected in previous studies. An interaction of the fiber length with fiber strength was identified in a total of 13 pairs with each trait in two generations. The study concluded with five important chromosome segments having important effects on fiber quality and yield (Song et al., 2017).

Fiber quality and yield-associated genes were identified by constructing a high-density genetic map using SLAF-seq, while incorporating 239 RILs derived from LMY22 (high-yielding *G. hirsutum* L. cultivars) x LY343 (superior fiber quality germplasm with *G. barbadense* L. introgressions) (Wang F. et al., 2019). The resulted genetic map was spanned over 3,426.57 cm which included 3556 SLAF-seq–based SNPs and 199 SSR marker loci. Under seven different environments, 67 QTLs for fiber quality and 37 QTLs for fiber yield were detected making a total of 104 QTLs. It was noted that on chromosome 12 with 19 QTL clusters, 66 QTLs were co-located, and 24 stable QTLs were determined under more than three different environments. The role of the genomic component LY343 was also investigated on fiber quality-related traits which indicated the superior contribution of *G. hirsutum* races than *G. barbadense* (Wang F. et al., 2019).

As discussed previously, the economic value of cotton relates to the quality of fiber, and enzymes play a vital role in maintaining the fiber quality. In a study, it was reported that the laccase enzyme plays a crucial role in fiber elongation, lignification, and plant pigmentation (Balasubramanian et al., 2016). Some laccase genes have been identified genome-wide of cultivated *G. hirsutum* and its diploid progenitor (*G. arboreum* and *G. raimondii*) cotton species. The gene expression, enzymatic activity, and biochemical analysis have shown that the laccase enzyme is differentially expressed at different stages of fiber development, particularly at 25DPA (Days Post-Anthesis). During this study, it was noted that chromosomes 7 and 13 of *G. arboreum* contained the highest number of laccase genes, i.e., eight on each, indicating a significant contribution of laccase in fiber development (Balasubramanian et al., 2016).

A 63K Illumina Infinium SNP array was performed on 719 accessions of upland cotton for phenotyping and genetic variation analysis for their better understanding (Sun et al., 2017). Forty-six significant SNPs with five fiber quality traits were detected of the total 10,511 polymorphic SNPs distributed across 26 chromosomes. A total of 10,511 polymorphic SNPs were scattered across 26 chromosomes. Forty-six SNPs were found significantly associated with five fiber quality traits (Sun et al., 2017). The distribution of these significantly found SNPs across 15 chromosomes indicated their relatedness in 612 unique candidate genes with their involvement mainly in polysaccharide synthesis, signal transduction, and protein translocation. The significance of the study relates to the determination of two major haplotypes of the fiber length and strength across chromosomes Dt11 and At07, providing an insight into the genetic basis of fiber quality in *G. hirsutum* (Sun et al., 2017). In upland cotton, a recombinant inbred line was developed, and a consensus map was constructed using three types of markers under 17 environments Zhang et al., 2020). The study revealed some stable QTLs for fiber quality traits across chromosomes 4, 6, 13, 21, and 25 including chromosome 7. Out of the total, three stable QTLs were determined for the fiber length and strength, while two for fiber micronaire. Genomic and bioinformatics approaches are efficient to find and retrieve target QTL regions. The information on the fiber development mechanism can be deduced from the integration of transcriptome analysis and QTL mapping. Different reports and studies on fiber quality development can be further improved by making interspecific crosses and cotton genome information. Molecular markers, cotton genomes, and transcriptome information are vibrant to decipher and dissect fiber development mechanisms to cultivate superior varieties with improved fiber quality across chromosome 7 of *Gossypium* species.

A composite interval model (CIM) can be incorporated for QTLs or genes using molecular markers with several segregation patterns. The proposed model assists in the determination of QTLs, segregation pattern, the estimation of their position, and the interference of their linkage phase with the markers. It has been reported that a simulation study shows the significance of the model (Gazaffi et al., 2014).

## Conclusion and Future Prospects

Several differentially expressed genes associated with fiber quality are reported using the marker-assisted selection strategy. Decades ago, many QTLs both in diploid and tetraploid cotton species have been reported in different populations having different characteristics from their parents. Genome sequencing provided breeders and scientists to develop high-density genetic maps based on SSR and SNPs, phenotypic and genotypic interactions, QTL identification, and fine mapping for candidate gene selection across chromosomes. The cotton genome, DNA markers, and transcriptome studies reveal the significant role of different chromosomes harboring genes associated with the fiber quality, as described in Tables. It was noticed that chromosomes 3, 7, 9, 11, and 12 associated with sub-genomes A and D contribute significantly to fiber quality. However, based on this information, our laboratory is focusing on chromosome 7 in dissecting the mechanisms of fiber quality improvement. Furthermore, RT-qPCR and molecular cloning demonstrate the differential expression and structure of candidate genes located on chromosome 7. A great insight is expected into the genetic basis of fiber-related traits such as the fiber strength and fiber length on chromosome 7 for improvement in cotton. The contribution of chromosome 7 carrying candidate genes in fiber quality development requires more attention in the future for QTL fine mapping, marker-assisted selection (MAS) breeding, and ultimately transgene in *Arabidopsis* and subsequently in cotton.
